# A narrative review of advancing medical education through technology: the role of smart glasses in situated learning

**DOI:** 10.1186/s12909-025-06949-7

**Published:** 2025-03-10

**Authors:** Bahareh Ghavami Hosein Pour, Zahra Karimian, Nazanin Hatami Niya

**Affiliations:** 1 Virtual School and Center of Excellence in e-Learning, Student Research Committee, Shiraz University of Medical Sciences, Shiraz, Iran; 2 Department of e-Learning in Medical Sciences, Virtual School and Center of Excellence in e-Learning, Shiraz University of Medical Sciences, Shiraz, Iran; 3 Student Research Committee, Dezful University of Medical Sciences, Dezful, Iran

**Keywords:** Smart glasses, Augmented reality, Wearable technology, Medical students, Medical education

## Abstract

**Background:**

The integration of smart glasses technology in medical education has emerged as a promising approach to enhance medical education specially in clinical setting. Grounded in situational learning theory, smart glasses provide immersive experiences that allow students to engage with real-world clinical scenarios. The purpose of this study is to investigate the application and impact of smart glasses technology in medical education.

**Method:**

In this study, two databases, PubMed and ScienceDirect, were used from 2016 to 2024 with related keywords and specific terms. In the process of searching and collecting studies, we utilized the Statement of Preferred Reporting Items for Systematic Reviews and Meta-Analyses (PRISMA). The studies reviewed included full-text quantitative, qualitative, and review articles that addressed the impact of smart glasses. In the initial review, 123 articles were found, and after applying the inclusion and exclusion criteria, 13 articles were evaluated by the reviewers. Finally, 28 articles were selected for review.

**Result:**

The outcome of this review indicated that smart glasses significantly enhance procedural training by allowing students to observe live surgeries and interact with instructors in real-time. Additionally, the technology facilitated remote collaboration, enabling students to participate in training sessions regardless of geographical constraints.

**Conclusion:**

The integration of smart glasses into medical education presents a transformative opportunity to enhance traditional training methods and improve situated learning for students. By providing immersive and interactive learning experiences, smart glasses can enhance skill acquisition and foster a more engaging educational environment for future healthcare professionals.

## Introduction

In recent years, the use of wearable technologies across various fields, particularly in medical education, has increased significantly [1]. One of the primary challenges in medical education is ensuring patient safety. In this context, societal expectations for education have risen, emphasizing the need to prioritize this issue. Conversely, traditional teaching methods, often summarized as “see one, do one, teach one”, are no longer adequate for maintaining patient safety. Consequently, medical education professionals have increasingly turned to simulators, virtual environments, and technologies such as smart glasses [2].

Smart glasses are compact devices equipped with a display and a video camera. They are worn on the head and can connect to the Internet [3]. This technology enhances immersion and interaction in the real world for users. A systematic review revealed that smart glasses facilitate visual communication in a hands-free manner, which is essential for effective collaboration among medical teams [4]. The wearable technology of smart glasses has various applications in education, including the following: creating interactive and engaging learning experiences through augmented reality; recording and storing presentation content; enabling immediate and accurate information collection; transforming lectures into videos for review and retention; and storing essential information. Additionally, smart glasses facilitate distance learning and access to educational resources, assess and measure learner performance, and enhance understanding of the listener’s experience and needs by analyzing their behavior. They also measure attention and concentration levels during learning, improve the teaching process [5] and increase motivation and effectiveness in learning [6]. This technology promotes critical and analytical thinking [6] and fosters cooperative learning [7].

In numerous medical education programs, instructors frequently depend on traditional teaching methods that primarily focus on lectures. In conventional educational settings, participation and interaction tend to be minimal, resulting in a lack of retention of competency-based knowledge and skills. Furthermore, feedback in these environments is often delayed until after assessments, which restricts opportunities for immediate improvement. In contrast, wearable devices can offer real-time feedback during training sessions, enabling learners to adjust their techniques on the spot. This immediate reinforcement is essential for developing competencies in high-risk medical environments [8].

To achieve this goal, educational institutions must create authentic environments that allow students to engage in real-world scenarios within their educational programs. Given the challenges associated with providing genuine learning environments, it is essential to leverage digital technologies to facilitate these learning opportunities. Such opportunities enable trainees to acquire hands-on experience in managing practical cases [9]. In this context, Over et al. (2014) and Dunleavy and Dede acknowledge that learning facilitated by augmented reality (AR) technologies supports situated and constructivist learning theories [10].

Situational learning refers to a process in which learners acquire skills or procedures related to those skills within a specific context and social environment through the exchange of ideas [11–13]. Situated Learning Theory, developed by Jean Lave and Etienne Wenger in the late 1980s, emphasizes that learning is most effective when it occurs in authentic contexts where knowledge can be directly applied. This theory posits that knowledge is not merely acquired through traditional instruction but is constructed through participation in real-world activities and social interactions within a community of practice [12–14].

Smart glasses technology, grounded in situated learning theory, can serve as an effective tool for learning in real-world environments [15].

By providing immediate access to information and facilitating social interactions, this technology promotes deeper learning in medical education and assists learners in applying their skills in practical situations. In addition to enhancing procedural skills, smart glasses also facilitate remote training and collaboration.

A case study investigating the use of smart surgical glasses during cleft surgery found that these devices provided an improved view of the surgical field and enabled remote assistance for local surgical residents. This capability is especially crucial in low-resource settings, where access to specialized training may be restricted [16]. Therefore, the instruction of surgical procedures is one of the key applications of these technologies, as the competence expected from learners is closely linked to the extent to which their educational experiences mirror real-world scenarios. However, due to the continuous advancement of these technologies, instructors—including surgeons—have identified several limitations despite the positive outcomes observed in limited trials. These limitations include high costs, concerns regarding patient privacy, and the insufficient skills of both instructors and learners [17]. However, this technology, which enables access to virtual images of the human body, is still utilized in surgical training. This allows learners to gain substantial experience before participating in real-life scenarios [18]. Additionally, the effectiveness of smart glasses in training for paediatrics procedures has been investigated, with results indicating an increase in success rates and a reduction in the time taken to complete the procedure on the first attempt [19]. Therefore, the necessity and importance of integrating smart glasses technology into medical education cannot be overstated. As this technology continues to evolve, it promises to revolutionize the delivery of medical education, making it more accessible, interactive, and effective for future healthcare professionals. This study was conducted to investigate the impact of smart glasses on medical education, with a focus on their effectiveness in enhancing skill acquisition. This research seeks to explore the following inquiry: How is smart glasses technology utilized in medical education, and what are its effects, particularly from the viewpoints of students and faculty?

## Literature review

### Situated learning

The theory of situational learning emphasizes that the learning process should commence in practical conditions and contexts [11, 12]. This theory posits that active participation of the learner in authentic learning environments is essential, influenced by factors such as the type of activity, the environment, culture, and social interactions [12, 13, 14]. With this approach, learners can acquire knowledge and skills in real situations, enabling them to provide logical interpretations of their experiences. Wenger built on the situated learning theory, evolving it into a social learning theory. In this transformation, he reconceptualized learning as a means of fostering practice and enabling newcomers to develop their identities [14]. The term “practice” encompasses the collaborative interactions within communities, initiating the process through which knowledge is created and identities are reshaped. Conversely, “identity” represents the outcome of learning influenced by these practices. This concept is fluid and continuously evolves, affecting how learners understand themselves in relation to their surroundings. Furthermore, practice is reinforced through the “negotiation of meaning,” which involves assigning significance to experiences acquired through active participation in one’s environment [20].

### Key components of situated learning

#### Authentic contexts

Learning should occur in environments that closely resemble the contexts where the knowledge will be applied. This principle is emphasized in various educational frameworks, highlighting the importance of real-world relevance in learning activities [21, 22]. Authentic learning tasks are designed to reflect the practices of specific disciplines, ensuring that students engage with material that resonates with their lived experiences [22, 23].

*Legitimate Peripheral Participation (LPP)*: This concept describes how newcomers gradually become integrated into a community of practice by starting with simple tasks and advancing as they gain experience. LPP illustrates learning as a social process achieved through participation in community activities [12]. It emphasizes the importance of mentorship and guided interactions with more experienced members to facilitate skill acquisition and identity formation within the community [20].

#### Community of practice

Learning is framed as a social activity occurring within groups that share common interests or professions. These communities foster collaborative interactions that enhance collective knowledge and understanding [12–14, 20, 23]. The dynamics within these groups promote shared learning experiences and contribute to the development of professional identities among participants [12, 20, 23].

### Situated learning and emergent technologies

Situated learning theory, emphasizes that learning is inherently tied to the context in which it occurs. This theory has significant implications for the integration of new emergent technologies in educational settings [12]. The integration of new technologies into practical learning environments enhances the learner’s ability to understand the subject matter in a more meaningful way. Ultimately, achieving situational learning necessitates the implementation of thoughtful and experiential teaching and learning strategies that can transform the teaching-learning process [24]. This technology integrates information into both virtual and real learning environments, allowing learners to engage in a blended experience that enhances the validity of cognitive activities. Learners can intuitively visualize the concepts being taught, thereby increasing their interaction with the surrounding elements. Through this reciprocal influence, both learners and learning environments are continuously evolving, enabling a deeper understanding and mastery of the content being studied [25].

As mentioned, understanding contexts is crucial in situated learning, as they provide environments that closely mirror real-world applications. With the advent of new technologies, educators can create immersive learning experiences that simulate these authentic contexts. For instance, virtual reality (VR) and augmented reality (AR) can replicate real-life scenarios where learners can practice skills in a safe yet realistic setting. This aligns with the notion that effective learning occurs when students engage with material relevant to their lived experiences, enhancing their ability to apply knowledge in practical situations [20, 21]. Also, emerging technologies facilitate this process by providing platforms for interaction and collaboration among learners and experienced practitioners. For example, online forums, collaborative tools like Google Docs, and social media platforms enable learners to participate peripherally by observing discussions and gradually contributing as they gain confidence and expertise. This digital engagement mirrors traditional apprenticeship models but expands access to diverse communities of practice beyond geographical limitations [12]. The concept of a Community of Practice is also enriched by new technologies. Online learning environments allow for the formation of communities where individuals with shared interests can collaborate regardless of location. Tools such as discussion boards, webinars, and video conferencing facilitate ongoing dialogue and knowledge sharing among participants. This collaborative aspect not only enhances individual learning experiences but also fosters a collective understanding and development of professional identities within the community [20, 22]. In summary, situated learning theory provides a robust framework for understanding how new emergent technologies can enhance learning experiences. By creating authentic contexts, supporting legitimate peripheral participation, and fostering communities of practice through digital platforms, educators can leverage technology to facilitate deeper engagement and more meaningful learning outcomes. This approach aligns with the evolving needs of learners in a rapidly changing technological landscape, ensuring that educational practices remain relevant and effective.

### Smart glasses technology

Smart glasses are wearable devices that incorporate technology into a pair of eyeglasses, allowing users to access digital information seamlessly integrated into their field of vision. They typically feature a miniature display system that projects content such as text, images, and videos directly onto the lenses or through a transparent screen. This integration creates an augmented reality experience where the physical and digital worlds converge [26, 27]. They are equipped with various technologies that enhance the learning experience. Here are some key features and benefits of smart classrooms.

- *Interactive Learning Tools*: Smart glasses utilize interactive tools that facilitate engaging learning. These technologies allow teachers to present information in dynamic ways, making learning more focused and enjoyable for students [28–32].
- *Collaborative Learning*: The design of smart glasses promotes collaboration among students. Interactive displays facilitate group work and discussions, enabling students to share ideas and learn from one another effectively [33, 34].
- *Sense of presence*: Smart glasses technology plays a significant role in enhancing learners’ experiences by facilitating a sense of presence, immediacy, and immersion, as well as visualizing elements that are otherwise invisible [35, 36].
- *Remote Learning Capabilities*: With advancements in technology, smart glasses can accommodate remote learning. Students who cannot attend in person due to illness or other reasons can participate in classes virtually, ensuring they do not miss out on learning opportunities [37].
- *Personalized Learning Experiences*: Smart classrooms often utilize software that allows for personalized learning paths tailored to individual student needs. This customization helps enhance student engagement and commitment to learning [38, 39].

Smart glasses technology is a transformative advancement in education, enhancing learning experiences by seamlessly integrating digital information into the user’s field of vision and creating augmented reality environments. Key features, such as interactive learning tools and collaborative capabilities, promote engagement and immersion, allowing students to visualize complex concepts. This integration not only improves accessibility but also prepares learners for a technology-driven future. Despite challenges related to affordability and adoption, smart glasses hold significant potential to revolutionize teaching and learning, offering exciting opportunities for educators and students in an evolving educational landscape.

Despite their potential benefits, smart glasses have not been widely adopted in educational settings due to the absence of clear educational frameworks. Current training methods often fail to align with the capabilities offered by smart glasses. For effective integration, educators require training on how to utilize these tools effectively within their curricula [40]. Research indicates that teacher acceptance is crucial for the successful implementation of smart glasses in education. Factors influencing acceptance include perceived usefulness, compatibility with existing teaching methods, and the availability of support for educators. If these factors are not addressed, the integration of smart glasses in classrooms may be limited [41]. Therefore, the need for an effective framework for formulating teaching strategies with technology and its acceptance is necessary.

## Methods

### Study design

This research is a narrative review that examines articles related to smart glasses and their connection to situated learning in medical education. It focuses on synthesizing and analyzing the existing literature regarding how smart glasses technology has been integrated into educational contexts, particularly in enhancing situated learning experiences. Given the rapidly evolving nature of smart glasses technology, this review encompasses articles published between 2016 and 2024, allowing for a comprehensive exploration of the latest advancements and applications in the field.

### Search strategy

A comprehensive search strategy was employed to review and extract studies investigating the impact of smart glasses in medical education. Search terms included a combination of keywords such as ‘smart glasses,’ ‘augmented reality,’ ‘wearable technology,’ ‘medical students,’ ‘medical education,’ and ‘situated learning.’ Boolean operators (AND, OR) were used to refine the search results in PubMed and ScienceDirect. The search was limited to articles published between 2016 and 2024. Please see Table 1.

It is noteworthy that the use of the term “situational learning” AND/OR “smart glasses” did not yield any articles directly addressing this topic; however, the results of this search and the articles mentioned indicate, indirectly, the use of this technology in situational learning.

**Table 1:** MeSH terms and keywords

PICO (s)	Keyword/ MeSH	Combine Search
**Population**	Medical student, Medical teacher	(“Medical student” OR “Medical Teacher”)
**Intervention**	Smart glasses, Augmented reality, Wearable technology	(“Smart Glasses” OR “Augmented Reality” OR “Wearable technology”)
**Comparison**	-	-
**Outcome**	Educational Outcomes (e.g., Skills Acquisition)	(“Educational outcomes” OR “Skills acquisition”)

### Study selection

The selection criteria for this review focused on ensuring the relevance and quality of studies examining the impact of smart glasses on medical education. Articles included in the review were required to be directly related to the effects of smart glasses within this educational context, specifically excluding any that did not address this relationship. The study design was limited to the full text of qualitative, quantitative, and experimental studies, as well as review articles that addressed the impact of smart glasses. Editorial pieces, books, theses, and letters were excluded to maintain a focus on empirical research (Table 2).

**Table 2:** Inclusion and exclusion criteria

Criteria Type	Inclusion Criteria	Exclusion Criteria
**Study Relevance**	Articles that directly related to the effect of smart glasses on medical education	Articles that don’t address the effect of smart glasses on medical education
**Study Design**	Qualitative/ quantitative/ experimental studies	Editorials, Books Theses, Letter
**Quality Assurance**	Peer-reviewed full texts articles to ensure the quality of information.	Non-peer-reviewed articles, those lacking rigorous methodology, and abstracts
**Screening Process**	Titles and abstracts screened for relevance; full texts of potentially relevant articles reviewed. Included dates from 2016 to 2024	Articles that do not meet the inclusion criteria after full-text review

The screening process involved an initial review of titles and abstracts for relevance, followed by a thorough examination of the full texts of potentially relevant articles. This review was confined to articles published between 2016 and 2024, with any that did not meet the established criteria being excluded after full-text analysis. This systematic approach aimed to provide a comprehensive and reliable overview of the current literature on the role of smart glasses in medical education.

### Data extraction

**Fig. 1:**
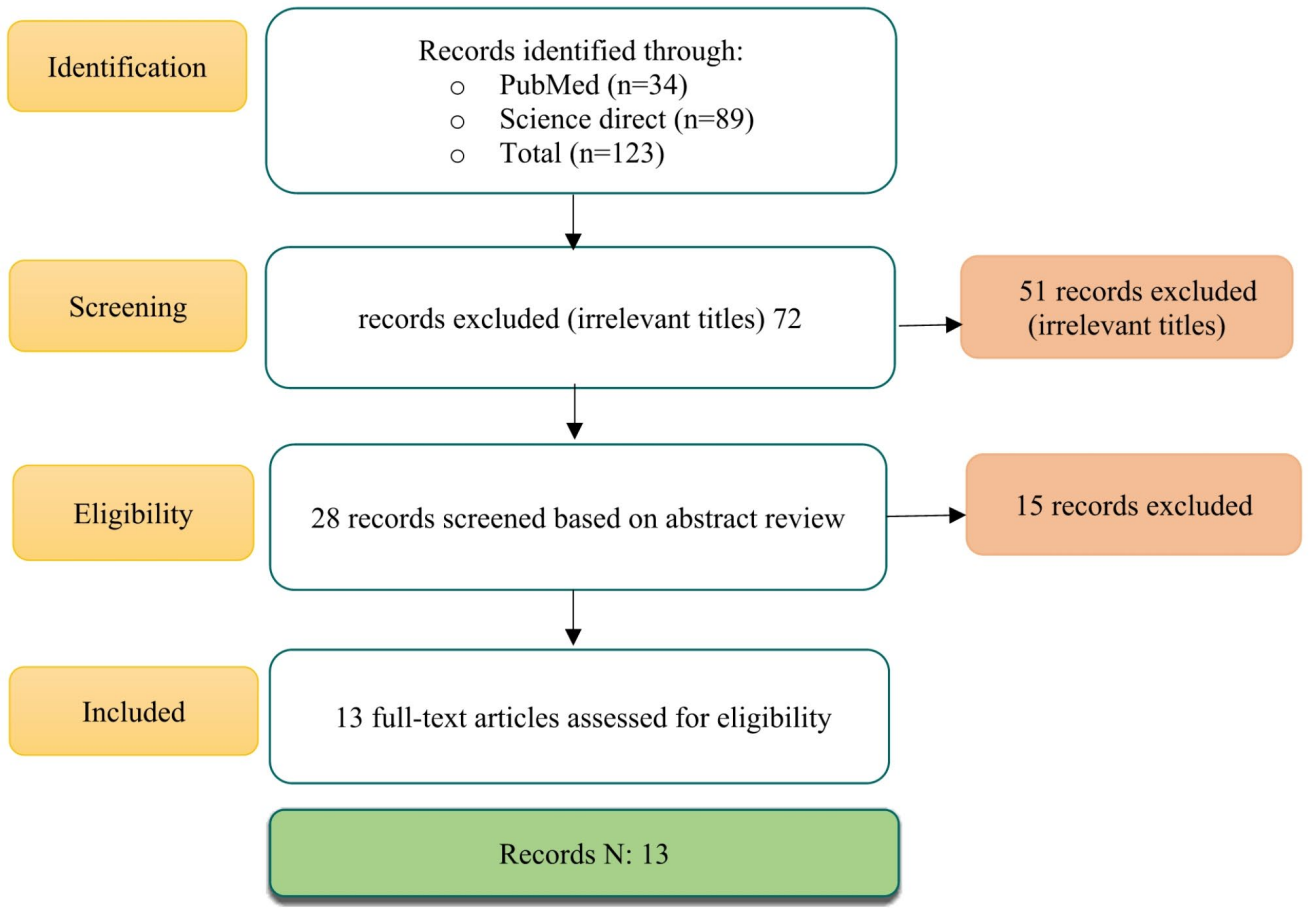
PRISMA flow-diagram for systematic reviews

In the data extraction process, three reviewers evaluated the studies using the standard Preferred Reporting Items for Systematic Reviews and Meta-Analyses (PRISMA) extraction checklist to ensure the consistency and comprehensiveness of the collected information. This checklist includes features such as the study title, author information, year of publication, study objectives, methodology, and key findings, providing a summary of the main points (Fig. 1).

### Quality assessment process

We prioritized methodological rigor by implementing a comprehensive quality assessment of the included studies. This process involved the following key steps:

1. **Selection of Assessment Tools**: We selected the Critical Appraisal Skills Programme (CASP) for evaluating randomized controlled trials. CASP provides a structured framework to assess the validity, results, and relevance of the studies. For non-randomized studies, we employed ROBINS-I (Risk Of Bias In Non-randomized Studies - of Interventions), which helps identify and evaluate biases in the design, conduct, and reporting of these studies.
2. **Assessment Criteria**: The assessment criteria included:

- **CASP**: The assessment focuses on several criteria, including clarity of the research question, appropriateness of the study design, recruitment of participants, control of variables and bias, and validity and reliability of the results.
- **ROBINS-I**: This tool evaluates confounding factors, selection bias, misclassification of interventions, deviations from intended interventions, missing data, measurement of outcomes, and reporting bias.

3. **Process of Evaluation**: Each included study was independently appraised by two reviewers to ensure objectivity and reduce bias. Discrepancies in assessments were resolved through discussion or by consulting a third reviewer. The quality ratings from both CASP and ROBINS-I were summarized, providing a clear overview of the methodological strengths and weaknesses of each study.
4. **Reporting Findings**: The results of the quality assessments were documented in our review. We included a table summarizing the quality ratings, which highlights the methodological quality of the studies and informs readers about the reliability of the findings.
5. **Implications for Future Research**: By conducting this thorough quality assessment, we aim to enhance transparency and provide a robust foundation for future research in this area.

## Result

Table 3 presents a comprehensive summary of recent studies examining the effectiveness of smart glasses in medical education. In this investigation, a total of 13 articles were identified through a search of two databases. The studies encompass observational research, systematic reviews, and randomized controlled trials, highlighting key findings related to skill acquisition, student satisfaction, and the overall impact of smart glasses on educational outcomes. Among the reviewed articles, nine examined the impact of using smart glasses, specifically Google Glass, on clinical skills, surgical procedures, and the enhancement of educational quality across various stages and learning environments, with a focus on student populations. Two articles investigated the effectiveness of these glasses in empowering faculty and improving their performance in training and clinical evaluations. One study assessed students’ perceptions regarding the use of this technology. Additionally, one article discussed the application of smart glasses as a supportive tool in teaching nursing skills (Table 3).

**Table 3:** Summary of the reviewed studies

Author(s)	Publication Year	Goal	Field	Methodology	Findings
Muroi et al. [42]	2024	• Investigating the effect of using educational content based on smart glasses for reflective learning (OSCE) from the perspective of patients and students	Reflective learning in OSCE	Intervention study	• Education based on smart glasses can be effective in the Ascii tests due to its ease of use
Araújo et al. [43]	2024	• To examine the incorporation of wearable electronic devices in the education of undergraduate nursing students.	Nursing education	Systematic Review	• Smart glasses useful and feasible as learning tools in nursing education, increasing nursing students’ motivation, confidence and satisfaction and can contribute to developing competencies required for the professionalism of undergraduate nursing students
Willis et al. [44]	2024	• Investigating the use of assisted reality technology to create a smart classroom in intensive care	Intensive care	Survey study	• Using assisted reality technology in smart class on intensive care was well received by students and educators
Smit et al. [16]	2024	• Assessing the usability of smart surgical glasses during cleft surgery and explore their potential in remote surgical education and collaboration	Cleft surgery	Cross-sectional survey study	• The smart glasses had several significant advantages over conventional on-site education, such as facilitating a better view of the surgical field and providing possibilities for remote interaction
Sridhar et al. [45]	2023	• Investigating the effectiveness of smart glasses technique in teaching procedural and surgical skills in medical students	Procedural and surgical skills	E-survey	• Validation of the effectiveness of smart glasses for clinical professors to use in teaching students and caring for patients
Reed et al. [46]	2023	• Investigating the use of smart glasses to empower faculty members in Objective Structured Teaching Exercises (OSTEs)	OSCE	Survey study	• Use of smart glasses during an OSTE on giving feedback was a non-distracting and positive experience. SG provided affective feedback otherwise not perceived from a standard MWC
Romare & Skär [47]	2023	• Overview of the usability and feasibility of smart glasses in nursing education. In addition, this study will highlight nursing students’ experiences of using smart glasses in learning situations	Nursing education	Scoping review	• Smart glasses have been used in a variety of learning situations in nursing education and enabled new learning situations. Students found smart glasses beneficial for their learning and smart glasses motivated and engaged students in the learning situation
Lin et al. [48]	2022	• Investigating the level of satisfaction and self-evaluation of medical students with the technology of smart glasses in the tube endotracheal insertion and central venous catheterization in clinical practice	Endotracheal insertion and central venous catheterization	Observational study Non-equivalent control-group Pre-and post-test	• The effectiveness of smart glasses in the education of medical students and the increase in satisfaction with the teaching method and self-efficacy have been evaluated
Lareyre et al. [49]	2021	• Investigating concepts related to smart glasses technology and potential applications and current limitations for its use in vascular surgery	Vascular surgery	Literature review	• The studies conducted so far show the potential of HMD and smart glasses for teaching surgery, anatomical sciences and distance learning
Carrera et al. [50]	2019	• Investigating use of Google glasess in Graduate medical education in the clinical learning environment, its use for resident supervision and education, and its clinical utility and technical limitations	Clinical education	Systematic Review	• Overall experience with use of Google glasess in Graduate medical education is generally positive • Evaluation of Google Glass in surgical specialties to teach or monitor surgical skills and use it for video conferencing
Dickerson et al. [51]	2019	• Investigating assess the feasibility and effectiveness of using a point-of-view video camera (Google Glass) to improve the surgical skills education of orthopedic surgery residents	Surgical skills education of orthopedic surgery	Randomized Controlled Trial	• Validation of the potential and useful role of video training with Google Glass in orthopedic surgery training
Wei et al. [52]	2019	• To conduct a systematic evaluation of the literature on the feasibility and acceptability of using Google Glass in surgical settings and to assess the potential benefits and limitations of its application	Surgical settings	Systematic review	• Google Glass can be used for surgical training, and several studies have confirmed the potential of this technology for patient education, consultation, and monitoring
Marschollek [53]	2016	• Investigate the feasibility and perceived usefulness of advanced smart glasses for an exemplary, specific activity in nursing training	Nursing education	Semi-structured interview and survey (Mixed Method)	• Nursing trainees found smart glasses technology useful for supporting education in nursing care

### Field

Among the 13 articles, the use of smart glasses was reported in surgical education (6 articles), nursing education (3 articles), OSCE testing (2 articles), intensive care (1 article), and clinical education (1 article).

### Type of articles

Out of the 13 articles, 6 were review articles, 3 were intervention and experimental studies, 4 were survey and cross-sectional studies, and 1 was a mixed-methods study (quantitative and qualitative).

### Impact of smart glasses

In all of these articles, the evaluation of smart glasses technology has yielded positive results. These technologies are increasingly being adopted and embraced due to their ability to enhance teaching and learning opportunities while overcoming the limitations of traditional education. This study synthesizes various research findings to highlight the growing recognition of smart glasses as a valuable educational tool. It emphasizes the multiple potentials of smart glasses in shaping the future of medical education and ultimately improving patient care outcomes. By examining these insights, we can gain a deeper understanding of the evolution of this technology.

### Key findings

The use of smart glasses in education has proven to be highly effective, particularly in Objective Structured Clinical Examinations (OSCEs), largely because of their user-friendly nature [42]. These devices have shown to be valuable and practical tools for learning in nursing education, resulting in heightened motivation, confidence, and satisfaction among students [43]. The integration of augmented reality technology in smart classrooms, especially for training in intensive care, has received positive feedback from both students and instructors [44]. Additionally, smart glasses provide significant benefits compared to traditional teaching methods, such as improved visibility during surgical procedures and enhanced opportunities for remote collaboration [16]. Their effectiveness in teaching procedural and surgical skills to clinical professors has been confirmed [45], and their application in Objective Structured Teaching Exercises (OSTEs) has fostered a conducive environment for faculty feedback without distractions [46]. Ultimately, incorporating smart glasses into medical education not only enhances the learning experience but also equips students to tackle real-world clinical challenges.

## Discussion

The integration of smart glasses into medical education has garnered significant attention in recent years, as evidenced by the diverse range of studies highlighted in the table. These studies collectively underscore the potential of smart glasses to enhance learning experiences for medical students and healthcare professionals across various disciplines. One prominent theme that emerges from this body of research is the effectiveness of smart glasses in improving procedural and surgical skills; Because our study focused on the use of smart glasses in teaching clinical and surgical procedures, the review of articles selected with this filter also demonstrates the effectiveness of these glasses in such courses. For instance, Sridhar et al. (2023) validated the use of smart glasses for clinical professors in teaching and patient care, demonstrating their utility in procedural skill acquisition [45]. Similarly, Lin et al. (2022) reported increased satisfaction and self-efficacy among medical students when using smart glasses for critical procedures like endotracheal intubation and central venous catheterization. These findings align with the principles of situational learning theory, which emphasizes the importance of context and real-world application in the learning process [48]. Moreover, studies such as those by Araújo et al. (2024) and Marschollek (2016) highlight how smart glasses can enhance motivation, confidence, and engagement among nursing students [43, 53] The incorporation of wearable technology into nursing education has shown to be beneficial not only for skill development but also for fostering a deeper understanding of patient care contexts. This is particularly relevant in today’s healthcare landscape, where hands-on experience is crucial for developing competencies. The literature also reflects a growing consensus on the feasibility and positive reception of smart glasses among educators and students alike. For instance, Reed et al. (2023) found that using smart glasses during Objective Structured Teaching Exercises (OSTEs) provided valuable feedback without being distracting, thereby enhancing the teaching experience [46]. Furthermore, studies exploring Google Glasses in graduate medical education reveal a generally positive experience, indicating that these technologies can effectively support resident supervision and education [50]. Therefore, creating suitable educational environments enhances student learning and boosts the professional self-confidence of medical students regarding their theoretical, practical, and clinical skills. Consequently, the adoption of these technologies is well-received, fostering a positive atmosphere among learners [44].

The integration of smart glasses in medical education presents promising opportunities for enhancing learning outcomes; however, several challenges must be addressed to ensure their effective implementation [49]. Technical limitations, such as battery life and connectivity issues, pose significant hurdles, particularly in high-acuity environments where uninterrupted use is crucial. Additionally, potential distractions from the technology itself can hinder the learning process such as technological constraints and insufficient organisational support, were barriers to sustained integration. necessitating adequate training for both educators and students to maximize the benefits of this innovative tool [54–57]. Moreover, concerns regarding data privacy and security remain paramount. As highlighted by Lareyre et al. (2021), there are specific limitations in applying smart glasses within certain medical fields, including vascular surgery, which may affect their broader adoption [49]. Addressing these challenges through ongoing research and development will be crucial in realizing the full potential of smart glasses in medical education.

**Practical Suggestions for Integration.**

1. Curriculum Enhancement: Integrate smart glasses into educational programs by creating dedicated modules that leverage this technology for skill enhancement.
2. Faculty Training: Create training initiatives for educators to effectively implement smart glasses in their teaching and assessment practices.
3. Pilot Programs: Implement pilot programs to assess the effectiveness of smart glasses across different educational settings prior to widespread adoption.
4. Feedback Systems: Set up feedback systems for both students and educators to evaluate the influence of smart glasses on educational outcomes.

These suggestions are designed to support the incorporation of smart glasses in medical education, enabling both learners and instructors to fully benefit from this cutting-edge technology.

## Conclusion

In conclusion, the reviewed studies highlight the significant potential of smart glasses to transform medical education by enhancing learning outcomes across various healthcare disciplines. These technologies have been shown to improve procedural skills, increase student satisfaction and self-efficacy, and foster collaborative learning environments. As we move forward, integrating smart glasses into medical curricula can create more immersive and effective educational experiences. Future research should focus on overcoming challenges related to technology integration and exploring innovative applications in diverse training scenarios. By leveraging the capabilities of smart glasses, educators can better prepare healthcare professionals for the complexities of clinical practice, ultimately leading to improved patient care in an increasingly technological healthcare landscape.

## Data Availability

Yes, the data analyzed during the current study are available from the corresponding author on reasonable request.
